# *APOBEC3A/B* deletion polymorphism and endometrial cancer risk

**DOI:** 10.1002/cam4.5448

**Published:** 2022-11-16

**Authors:** Nigar Sofiyeva, Camilla Krakstad, Mari K. Halle, Tracy A. O'Mara, Pål Romundstad, Kristian Hveem, Lars Vatten, Per E. Lønning, Liv B. Gansmo, Stian Knappskog

**Affiliations:** ^1^ K.G. Jebsen Center for Genome‐Directed Cancer Therapy, Department of Clinical Science University of Bergen Bergen Norway; ^2^ Department of Oncology Haukeland University Hospital Bergen Norway; ^3^ Department of Clinical Science, Centre for Cancer Biomarkers University of Bergen Bergen Norway; ^4^ Department of Obstetrics and Gynaecology Haukeland University Hospital Bergen Norway; ^5^ Cancer Program QIMR Berghofer Medical Research Institute Brisbane Australia; ^6^ Department of Public Health, Faculty of Medicine Norwegian University of Science and Technology Trondheim Norway; ^7^ K.G. Jebsen Center for Genetic Epidemiology, Department of Public Health, Faculty of Medicine Norwegian University of Science and Technology Trondheim Norway

**Keywords:** APOBEC, deletion, endometrial cancer, risk

## Abstract

**Background:**

A common 30 kb deletion affecting the *APOBEC3A* and *APOBEC3B* genes has been linked to increased *APOBEC* activity and APOBEC‐related mutational signatures in human cancers. The role of this deletion as a cancer risk factor remains controversial.

**Materials and Methods:**

We genotyped the *APOBEC3A/B* deletion in a sample of 1,470 Norwegian endometrial cancer cases and compared to 1,918 healthy controls. For assessment across Caucasian populations, we mined genotypes of the SNP rs12628403, which is in strong linkage disequilibrium with the deletion, in a GWAS dataset of 4,274 cases and 18,125 healthy controls, through the ECAC consortium.

**Results:**

We found the *APOBEC3A/B* deletion variant to be significantly associated with reduced risk of endometrial cancer among Norwegian women (OR = 0.75; 95% CI = 0.62–0.91; *p* = 0.003; dominant model). Similar results were found in the subgroup of endometrioid endometrial cancer (OR = 0.64; 95% CI = 0.51–0.79; *p* = 3.6 × 10^−5^; dominant model). The observed risk reduction was particularly strong among individuals in the range of 50–60 years of age (OR = 0.51; 95% CI = 0.33–0.78; *p* = 0.002; dominant model). In the different populations included in the ECAC dataset, the ORs varied from 0.85 to 1.05. Although five out of six populations revealed ORs <1.0, the overall estimate was nonsignificant and, as such, did not formally validate the findings in the Norwegian cohort.

**Conclusion:**

The *APOBEC3A/B* deletion polymorphism is associated with a decreased risk of endometrial cancer in the Norwegian population.

## Graphical abstract

The APOBEC3A/B deletion variant was significantly associated with reduced risk for endometrial cancer among Norwegian women. Although 5 out of 6 populations in the large ECAC data set revealed ORs <;1.0, the overall estimate was non‐significant and did not validate the findings in the Norwegian cohort.

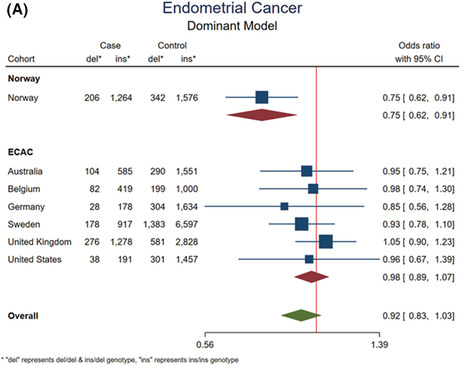

## 1 INTRODUCTION

Cancer of corpus uteri is one of the most common cancers among females.^1^ Besides environmental and reproductive factors leading to hormonal imbalance, family history contributes to up to 5% of uterine cancers,^2^ including 2–3% linked to Lynch syndrome caused by alterations in DNA mismatch repair genes.^3^ In addition, low penetrance genetic variants have been identified: Meta‐analyses of data from genome‐wide association studies have reported several endometrial cancer risk loci, including *MYC*, *AKT1*, *CDKN2A*, *CDKN2B*, *WT1*, *NF1*, and other well‐established cancer‐related genes.^4^

Regarding somatic mutations, several large genomics reports have identified *TP53*, *PTEN*, *CTNNB1*, *PIK3CA*, *ARID1B*, *KRAS, POLE*, and *NRIP1* as main driver genes frequently somatically mutated in endometrial cancer.^5^, ^6^ However, the vast majority of somatic mutations found in the tumor genome are mutations believed not to be directly involved in cancer development but rather more neutral passenger mutations. Somatic mutations can be caused by exogenic factors, such as UV exposure and various carcinogens, or endogenous factors, such as base substitutions due to error‐prone polymerases and incorrectly or unrepaired DNA damage caused by impaired DNA damage response.^7^ Notably, several of these mutational processes may be identified by the characteristic pattern of mutations they cause, coined mutational signatures.^8^, ^9^

Two of these mutational signatures, single‐base substitution (SBS) 2 and SBS13, are associated with the APOBEC (apolipoprotein B mRNA editing catalytic polypeptide‐like) family of proteins.^8^, ^9^ The APOBEC enzymes bind to RNA and single‐stranded DNA and regulate their function by introducing nucleotide changes. Importantly, APOBEC activity targeting the host cell's own DNA may lead to mutations contributing to tumorigenesis.^10^ Signatures SBS2 and SBS13, indicating APOBEC activity, is linked with a high proportion of tumors in the breast, bladder, cervix, head, lung, and soft tissue cancers. Moreover, in the recently updated list of signatures, one doublet base substitution signature (DBS11) was also linked with APOBEC activity.^8^

A deletion of 30 kb (29,935) bp in chromosome 22 (position: Chr22: 389,625,11‐389,924,45; GRCh38.p13) results in loss of the 3′UTR of the *APOBEC3A* gene, a non‐coding region between *APOBEC3A* and *APOBEC3B*, and the entire coding region of the *APOBEC3B* gene.^11^ Thus, this deletion leads to the formation of a hybrid transcript consisting of the coding region sequence of *APOBEC3A* and the 3′UTR of *APOBEC3B*, resulting in a protein with an identical amino acid sequence APOBEC3A.^11^ Notably, the new chimeric *APOBEC3A/B* mRNA is more stable than the wild‐type *APOBEC3A* mRNA and, thus, through a higher overall translation, causes more DNA damage.^12^ This deletion variant is a common human deletion polymorphism with an overall worldwide allele frequency of 22.5%. Its percentage varies considerably between different ethnic populations, ranging from 0.9% to 92.9%, with the lowest rate in Africans and Europeans and the highest in Oceanic populations.^11^

Notably, numerous tumor types have been assessed for the potential association between the *APOBEC3A/B* deletion variant and cancer risk. However, such studies have mainly provided contradicting results (Table S1, ^13^, ^14^, ^15^, ^16^, ^17^, ^18^, ^19^, ^20^, ^21^, ^22^, ^23^, ^24^, ^25^, ^26^, ^27^).

Endometrial cancer typically shows high diversity in mutational signatures.^8^, ^9^ SBS2, one of the APOBEC‐related signatures, was found to be operative in 29% of uterine cancer samples and contributed to 3.3% of the overall mutations in endometrial cancer.^9^ Further, in a recent update, signature SBS13 was also reported in samples of uterine adenocarcinoma.^8^ So far, the potential association between the *APOBEC3A/B* deletion variant and the risk of endometrial cancer has not been formally assessed.

In the present study, we aimed to evaluate the *APOBEC3A/B* deletion as a potential risk modulating factor for endometrial cancer.

## 2 MATERIAL AND METHODS

### 2.1 Study population

The study cases in this case–control study were Norwegian women, among whom the great majority were Caucasians, admitted to Haukeland University Hospital from 2001 to 2009 with a diagnosis of primary endometrial cancer (*n* = 1470). As controls, we used the female fraction (*n* = 1918) of a previously reported sample set of 3749 healthy Norwegian individuals^23^, ^28^ initially enrolled in the population‐based Cohort of Norway (CONOR) study.^29^

The study was approved by the Regional Committees for Ethics in Medical Research (REK Midt‐Norge and REK Vest). All data were collected upon obtaining written informed consent from participants and analyzed according to the Norwegian guidelines for research on human samples.

### 2.2 Statistical power

Prior to our study, to the best of our knowledge, no formal assessments have been made regarding the potential impact of the *APOBEC3A/B* deletion variant on the risk of endometrial cancer. Thus, formal power estimates are challenging. However, we have recently performed a similar study on ovarian cancer, in which the estimates were based on the data from Qi et al.^25^ Here, with an odds ratio (OR) of 1.46, applying an alpha‐value of 0.05 and aiming for a 1‐beta of 0.9, this would require *n* = 860 in each comparison group (equal groups of cases and controls). For the present study on endometrial cancer, we had 1470 cases and 1918 controls available and therefore deemed the sample size as adequate.

### 2.3 *APOBEC3A/B* genotyping

DNA extracted from blood samples from all participants was analyzed for germline *APOBEC3A/B* using separate primer pairs and hybridization probes by quantitative polymerase chain reaction high‐resolution melting (qPCR‐HMR) curves on a LightCycler 480 II instrument (Roche Diagnostics, Basel, Switzerland) as previously described.^23^

For technical validation and analyses for samples failing genotyping, we performed genotyping of the SNP rs12628403 (Chr 22: 389,620,32). rs12628403 was genotyped as described previously^23^ using a custom‐made LightSNiP assay (TIB Molbiol GmbH, Berlin, Germany) according to the manufacturer's instructions. This SNP is located 478 bp upstream of the *APOBEC3A/B*‐deletion start point and is in strong linkage disequilibrium with the deletion in all investigated populations, including Norwegians.^13^, ^23^ Given the ethnic background of the study population and the strong linkage to rs12628403, this SNP was used as a surrogate marker to define the *APOBEC3A/B* deletion status. Out of the 1470 cases analyzed for *APOBEC3A/B* deletion, 520 were repeated with the SNP. In three cases, SNP analysis revealed a genotype not matching the expected genotype from the original deletion analysis. Thus, the observed recombination rate (fraction) was 5.8 × 10^−3^. This finding was in line with previous data in other sample sets.^26^ As such, the potential difference in results from *APOBEC3A/B* deletion analyses and rs12628403 analyses were considered negligible.

### 2.4 Mined dataset

We mined the data derived from the Endometrial Cancer Consortium (ECAC), including women of European ancestry from cancer centers in Australia, the United States, the United Kingdom, Germany, Belgium, and Sweden, for extended analyses. This dataset consisted of SNP data from women diagnosed with endometrial cancer and country‐matched controls, as described previously.^4^ Given that the genotyping of the ECAC samples was performed by SNP‐array (OncoArray), information about the status of the *APOBEC3A/B* deletion was not available per se. Therefore, in the ECAC data, we applied genotyping of the SNP rs12628403 (see paragraph above; “*APOBEC3A/B* genotyping”). Details of the genotyping are previously described by Amos et al.^30^ and O'Mara et al.^4^ In total, we assessed data from 4274 cases and 18,125 controls from the ECAC dataset.

### 2.5 Statistical analysis

Potential deviations from Hardy–Weinberg equilibrium (H–W) were assessed by calculating the expected genotype distribution based on the observed allele frequencies and comparing the output with the observed genotype distribution using the Chi‐square test for all sample cohorts. Genotype distributions were assumed in H–W‐equilibrium if nonsignificance was confirmed.

Possible associations between the *APOBEC3A/B* del variant and risk of endometrial cancer were evaluated by ORs with 95% confidence intervals (CIs) and chi‐square tests. Additional OR estimates were performed by logistic regression, adjusting for age or age groups, using the SNPassoc R package.^31^ Individual age information was available for the exploratory Norwegian sample sets, while age group information was available for the validation (ECAC) set. A surrogate meta‐analysis was performed to calculate weighted and pooled OR for cancer risk across populations using the *metan* command.^32^ ORs with 95% CI not spanning 1.0 were considered significant, unadjusted for multiple testing.

All statistical analyses were performed using the R studio (RStudio Team [2022]. RStudio: Integrated Development Environment for R. RStudio, PBC, Boston, MA URL http://www.rstudio.com/.) and STATA software v.17.0 (StataCorp. 2021. Stata Statistical Software: Release 17. College Station, TX: StataCorp LLC).

## 3 RESULTS

### 3.1 Distribution of *APOBEC3A/B* genotypes

In a series of healthy Norwegian female controls (*n* = 1918), we have previously reported 1,576 (82.1%) individuals to be heterozygous for the *APOBEC3A/B* insertion allele, while 323 (16.8%) were heterozygous and 19 (0.99%) were homozygous for the deletion allele (Table 1), resulting in a minor allele frequency (MAF) of 0.094, and genotype distribution in Hardy–Weinberg equilibrium (*p* > 0.59; Table 1).^23^ In the present study, genotyping the *APOBEC3A/B* deletion polymorphism in 1470 endometrial cancer (EC) patients, we also found the genotype distribution to be in Hardy–Weinberg (H–W) equilibrium (*p* = 0.78) with 1264 (85.9%) homozygous for the insertion allele, 199 (13.5%) heterozygous, and seven (0.48%) homozygous for the deletion allele (Table 1), resulting in a MAF of 0.072.

**TABLE 1:** Distribution of *APOBEC3A/B* genotype in cases and controls in Norwegian cohorts and sub‐cohorts of the ECAC consortium

Cohort / Country	*n*, Cases / Controls	Deletion Allele frequency (%)		Genotype *APOBEC3A/B* n (%)						Genotype distribution pairwise comparison to Norway^a^, *p*‐value		Hardy–Weinberg equilibrium, *p*‐value	
				*Cases*			*Controls*			*Cases*	*Controls*	*Cases*	*Controls*
		Cases	Controls	ins‐ins	ins‐del	del‐del	ins‐ins	ins‐del	del‐del				
**Norway**	1470 / 1918^b^	7.24	9.41	1264 (85.99)	199 (13.54)	7 (0.48)	1576 (82.16)	323 (16.84)	19 (0.99)	—	—	0.781	0.589
**ECAC**	4274 / 18,125	8.65	8.76	3568 (84.12)	673 (15.18)	33 (0.70)	15,067 (83.04)	2939 (16.28)	119 (0.69)	0.058	0.181	0.838	0.060
**Australia**	689 / 1841	7.91	8.26	585 (84.91)	99 (14.37)	5 (0.73)	1551 (84.25)	276 (14.99)	14 (0.76)	0.662	0.216	0.719	0.655
**Belgium**	501 / 1199	8.18	8.63	419 (83.63)	82 (16.37)	0 (0)	1000 (83.40)	191 (15.93)	8 (0.67)	0.094	0.497	0.046	0.732
**Germany**	206 / 1938	7.04	8.10	178 (86.41)	27 (13.11)	1 (0.49)	1634 (84.31)	294 (15.17)	10 (0.52)	0.986	0.078	0.982	0.407
**Sweden**	1095 / 7980	8.72	9.01	917 (83.74)	165 (15.07)	13 (1.19)	6597 (82.67)	1328 (16.64)	55 (0.69)	0.065	0.375	0.076	0.182
**United Kingdom**	1554 / 3409	9.30	8.80	1278 (82.24)	263 (16.92)	13 (0.84)	2828 (82.96)	562 (16.49)	19 (0.56)	**0.015**	0.181	0.896	0.114
**USA**	229 / 1758	8.52	8.93	191 (83.41)	37 (16.16)	1 (0.44)	1457 (82.88)	288 (16.38)	13 (0.74)	0.566	0.658	0.575	0.765
		*p* = 0.121^c^	*p* = 0.371^c^	*p* = 0.121^d^			*p* = 0.614^d^						

^a^ Comparison of genotype distribution between Norwegian cohorts and cohorts from ECAC consortium (Chi‐square test).

^b^ Data for Norwegian controls are previously published.^21^, ^27^

^c^ Comparison of allele distribution between all contributing country cohorts (Chi‐square test).

^d^ Comparison of genotype distribution between all contributing country cohorts (Chi‐square test).

For validation purposes, we mined available data from the ECAC consortium.^4^ Norwegian cases were excluded due to potential overlap with the main series of the present study. Since data for the *APOBEC3A/B*‐deletion were unavailable per se, we used the genotypes for the strongly linked SNP rs12628403 as a surrogate marker for the deletion status (see Materials and Methods “*APOBEC3A/B* genotyping” for concordance assessment). Here, we found the genotypes to be in H‐W‐equilibrium, both in endometrial cancer and control cohorts in the general consortium cohort (*p*‐value = 0.838 and 0.060, MAF = 0.086 and 0.088, respectively) and in all the individual populations (countries) contributing to ECAC, with the exception of Belgium, where the equilibrium was slightly skewed (*p* = 0.046; Table 1). The deletion allele frequency in the different countries across ECAC ranged from 0.070 to 0.093 in cases and 0.081 to 0.090 in controls (Table 1).

Evaluation of genotype and allele distribution in Norwegian and ECAC cohorts together revealed a homogenous data status in endometrial cancer cases (*p* = 0.121 and *p* = 0.121, respectively) and healthy controls (*p* = 0.614 and *p* = 0.371, respectively; Table 1; Figure S1).

### 3.2 *APOBEC3A/B* genotypes and endometrial cancer risk

**FIGURE 1:**
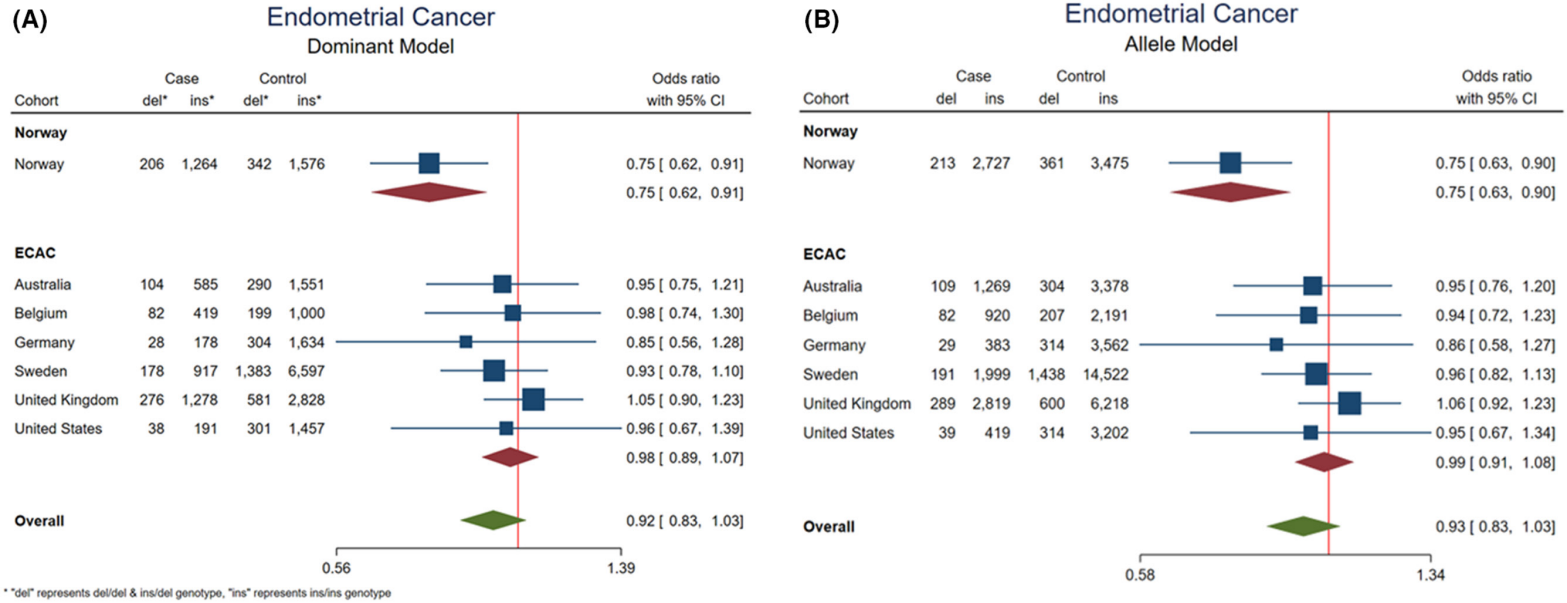
*APOBEC3A/B* deletion and risk of endometrial cancer. Forest plots illustrating ORs with 95% confidence intervals (CI) for endometrial cancer, related to the *APOBEC3A/B* deletion variant, applying A) the dominant model and B) the allele model.

To estimate the potential impact of the *APOBEC3A/B* deletion variant on endometrial cancer risk, we compared the frequency of the *APOBEC3A/B* genotypes among endometrial cancer patients (1470) to those of the healthy controls (1918). Applying individual models, not adjusted for multiple models, we found the *APOBEC3A/B* deletion variant to be significantly associated with reduced risk for endometrial cancer, applying both the dominant‐ and the allele‐models (OR = 0.75; 95% CI = 0.62–0.91; *p* = 0.003, OR = 0.75; 95% CI = 0.63–0.90; *p* = 0.0002, respectively; Figure 1, Table S2). The same trend was observed in the recessive model, although these data did not reach statistical significance, which could be stemmed from the low number of observations (OR = 0.48; 95% CI = 0.20–1.14; *p* = 0.089; Additional models are presented in Table S2).

In the mined data from the ECAC consortium, an overall assessment yielded an OR = 0.98; 95% CI = 0.89–1.07; *p* = 0.579 and OR = 0.99; 95% CI = 0.91–1.08; *p* = 0.726 in the dominant and allele models, respectively. Notably, stratifying the ECAC data into different populations (countries), all analyses per population revealed an OR below 1.0, except for the United Kingdom (Figure 1, Table S2). Meta‐analysis including all countries also resulted in a pooled OR indicating risk reduction, although not statistically significant (OR = 0.92; 95% CI = 0.84–1.02, *p* = 0.261; Figure 1).

### 3.3 Impact of *APOBEC3A/B* genotypes in endometrial cancer subtypes

We performed stratified analyses to assess the potential differential impact of the APOBEC3A/B deletion on different histological subtypes of endometrial cancer. In the subgroup of endometrioid endometrial cancer, we found a similar pattern as in the overall assessments, with a significantly reduced cancer risk in the Norwegian cohort both in the dominant and the allele models (OR = 0.64; 95% CI = 0.51–0.79; *p* = 3.6 × 10^−5^ and OR = 0.64; 95% CI = 0.52–0.79; *p* = 3.2 × 10^−5^, respectively; Table S3; Figure S2).

In the ECAC dataset, the findings were similar. There was a trend toward reduced risk for endometrioid endometrial cancer, although nonsignificant, for most countries, except for the United Kingdom and Belgium (dominant model). Pooled ORs from meta‐analysis revealed a decreased endometrioid endometrial cancer risk, although without statistical significance in either the dominant or the allele model (Figure S2).

No clear risk association was observed in subgroup analysis for cases with non‐endometrioid histology (Tables S4 and S5, Figure S3).

### 3.4 Interaction between *APOBEC3A/B* genotypes and age

Given our previous findings of an age‐related trend in the lung cancer risk among individuals with *APOBEC3A/B* deletion genotype,^23^ we performed subgroup analysis by age groups in the endometrial cancer cases and controls. The mean age of participants with endometrial cancer in the Norwegian cohort was 66, ranging from 28 to 98 years. Patients and controls from 50 to 80 years old were divided into age groups with a 10‐year cut‐off. The remaining individuals are classified as below 50 and above 80 years old.

**FIGURE 2:**
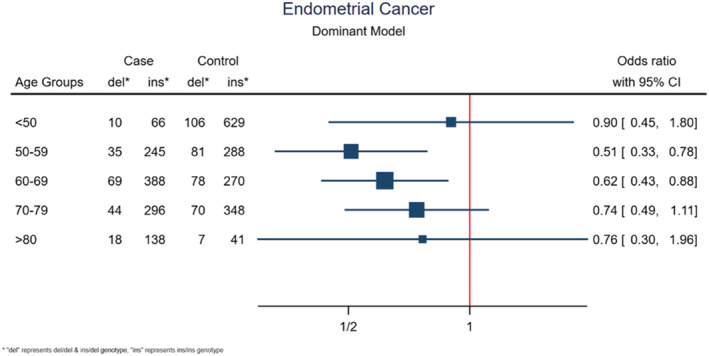
*APOBEC3A/B* deletion and age‐related risk of endometrial cancer. Forest plot illustrating ORs with 95% confidence intervals (CIs) for endometrial cancer, related to the *APOBEC3A/B* deletion variant, stratified in age intervals of cases and controls.

In the groups of 50‐ to 59‐ and 60‐ to 69‐year‐old individuals the *APOBEC3A/B* deletion was associated with a significantly reduced cancer risk (dominant model: OR = 0.51; 95% CI = 0.33–0.78; *p* = 0.002 and OR = 0.62; 95% CI = 0.43–0.88; *p* = 0.008, respectively; Figure 2 and Table S6). All other age groups also revealed slightly reduced risk linked to the deletion allele, although not reaching statistical significance. A similar result was found when restricting the analysis to cases with endometrioid histology (Table S6 and Figure S4). Notably, although the risk reduction was not prominent for the individuals below 50 years of age, for the four older age groups, there was a trend for the risk reduction being linked to young age, while this reduction shifted stepwise toward an OR of 1.0, with increasing age (Figure 2). However, an estimate of the trend across ranked age groups did not reach statistical significance (*p* = 0.321). A similar trend for age was not observed in the ECAC dataset (Figure S5).

Further, we went back to our main analyses and performed additional OR estimates by logistic regression adjusting for age. In the Norwegian sample set, these estimates also showed a significantly reduced endometrial cancer risk (dominant model: 0.70 (0.57–0.86), *p* = 7.2 × 10^−4^, allele model: 0.65 (0.42–1.00), *p* = 0.048) (Table S2). The same pattern was observed in the endometrioid endometrial cancer risk (dominant model: 0.63 (0.50–0.79), *p* = 4.1 × 10^−5^ and allele model: 0.61 (0.39–0.95), *p* = 0.026) (Table S3). Logistic regression analysis adjusting for age groups (available both for the Norwegian samples sets and ECAC) also resulted in estimates in line with our main calculations (Table S3).

## 4 DISCUSSION

Large genomics efforts over the last decade have provided in‐depth information about the landscape of somatic mutations in endometrial cancer. Thus, mutations in genes such as *TP53*, *PTEN*, *CTNNB1*, *PIK3CA*, *ARID1B*, *KRAS*, *POLE*, and *NRIP1* have been identified as driver genes frequently somatically mutated this malignancy.^5^, ^6^ Regarding germline factors associated with endometrial cancer, besides Lynch syndrome, these have largely been limited to low penetrance single nucleotide polymorphisms.^4^

While several studies have assessed the potential association between the *APOBEC3A/B* deletion variant and cancer development, the results are conflicting. For breast cancer, a high proportion of tumors (~90%) harbor both mutational signatures related to APOBEC activity, SBS2, and SBS13.^8^, ^9^ However, despite extensive research, conflicting results have been reported, with almost half of the studies being negative,^14^, ^19^, ^20^, ^21^, ^22^, ^23^ while conclusions from several meta‐analyses support an increased breast cancer risk in women carrying the *APOBEC3A/B* deletion variant.^22^, ^33^, ^34^ Bladder cancer is another cancer type with a high proportion of cases revealing APOBEC‐related mutational signatures. Here, pooled data show a decreased cancer risk for individuals with the deletion variant, although the number of studies is small.^22^ Studies investigating other tumor types are limited, and further studies are warranted for conclusive interpretations.^19^, ^22^, ^23^, ^35^ To the best of our knowledge, no studies have explored the potential risk of endometrial cancer associated with the *APOBEC3* deletion variant.

Our analysis of Norwegian women revealed the deletion variant to be associated with a reduced risk of endometrial cancer and the endometrioid endometrial cancer subtype. This association was confirmed across several models to compare genotypes and allele frequencies, unadjusted for the multiple models. Notably, our recessive model estimates were hampered by a very low number of individuals carrying the homozygous del/del genotype.

Aiming to validate our findings, we mined the ECACs GWAS study data, including >4000 cases and >18,000 healthy controls from six different populations (countries). Markedly, although all populations, except the United Kingdom, revealed ORs <1.0, the overall analysis did not reach significance and, as such, did not formally validate our findings in Norwegian samples. The frequency of the *APOBEC3A/B* deletion allele significantly varies between different populations. Women with European ancestry show a MAF of around 6%, while the corresponding crude MAFs are around 1%, 37%, and 93% in Africa, East Asia, and Oceania, respectively.^11^, ^36^ The different cohorts analyzed in the present study mainly consisted of Caucasian women, and we found the allele frequencies to be relatively homogenous between the seven countries (the Norwegian dataset and the six ECAC countries). Thus, although some of the differences seen in ORs could relate to the different ethnic compositions of analyzed populations, one must assume this effect to be limited.

It is worth mentioning that, while the Norwegian samples were analyzed at a different timepoint and mainly with a different method than the ECAC samples, previous data have indicated that the applied technologies provide consistent data. Also, in the present analyses, we compared the assay applications identifying the deletion variant per se versus an assay detecting SNP rs12628403 in linkage to the deletion and found the mismatch to be negligible. As such, it is unlikely that the difference observed between Norway and the overall ECAC data stems from methodological issues.

Regarding subtypes of endometrial cancer, it is worth noting that our results from the subgroup analysis in endometrioid endometrial cancer were in line with the overall results of general endometrial cancer risk. Thus, it may be that the overall results for endometrial cancer, in general, are primarily driven by the subgroup of endometrioid histology. Evaluation of the non‐endometrioid subtype of endometrial cancer revealed nonsignificant results. However, these assessments were underpowered as they were limited by small sample size and should be interpreted cautiously.

Our previous report showed an association between age and the impact of the *APOBEC3A/B* deletion in lung cancer and a similar trend in prostate cancers.^23^ Given these findings, we analyzed the possible interaction between age and the *APOBEC3A/B* deletion with respect to endometrial cancer risk. We observed a significantly reduced risk of endometrial cancer linked to the deletion variant in the group of individuals from 50 to 69 years of age. Markedly, we found a stepwise increase in OR in the 10‐year age groups from 50 years and upwards, although the trend did not reach significance. Interestingly, this stepwise increase is the opposite of what we previously observed for lung and prostate cancers, where there was a stepwise *decrease* in OR with increasing age.^23^ The reasons for this difference between the cancer types remain unknown. In our present study, the exception from the trend across age groups was the very youngest group of cases and controls, below 50 years of age. The same trend was seen within the subgroup of endometrioid cancers. The reason for this potential trend remains unknown, but it is worth noting that endometrial cancer is a hormone‐sensitive cancer type, and one may speculate that the difference in OR between the groups below or above 50 of age may be related to menopausal status.

The *APOBEC3A/B* deletion is strongly linked to APOBEC‐related mutational signatures^37^ and, as such, to processes contributing to tumor evolution and disease progression in established tumors. Based on this, one may assume that the deletion and a subsequent increased overall activity of APOBEC enzymes would increase cancer risk. However, it is worth noting that APOBEC activity is linked to anti‐viral and anti‐bacterial protection in non‐malignant cells. Given the link between infection and some cancer types (and the suspected link in other cancer types), it may therefore be that increased APOBEC activity may have a cancer‐protective function in some tissues. Whether this may be the case for endometrial cancer remains pure speculation, but it could provide an explanation for a reduced cancer risk linked to the *APOBEC3A/B* deletion.

Other functional polymorphisms in the *APOBEC* gene cluster have been identified. In particular the SNP rs1014971 has been linked to increased *APOBEC3B* expression and enrichment of APOBEC‐related mutational signatures in bladder cancer.^13^ Notably the *APOBEC3/B* deletion was not of importance in bladder cancer, while the opposite was the case for breast cancer. Thus, it seems there may be a tissue‐specific interplay between the impact rs1014971 and the *APOBEC3/B* deletion. The potential interaction between these two variants and the risk of endometrial cancer remains unknown.

## 5 CONCLUSION

The *APOBEC3A/B* deletion variant was significantly associated with reduced risk for endometrial cancer among Norwegian women. Although five out of six populations in the large ECAC dataset revealed ORs <1.0, the overall estimate was nonsignificant and did not validate the findings in the Norwegian cohort.

## AUTHOR CONTRIBUTIONS

**Nigar Sofiyeva:** Data curation (lead); formal analysis (lead); methodology (equal); writing – original draft (lead). **Camilla Krakstad:** Data curation (equal); resources (equal). **Mari Kylleso Halle:** Data curation (equal); resources (equal). **Tracy O'Mara:** Data curation (equal); formal analysis (equal); resources (equal). **Pål Romundstad:** Data curation (equal); resources (equal). **Kristian Hveem:** Data curation (equal); resources (equal). **Lars J Vatten:** Data curation (equal); resources (equal). **Per Eystein Lønning:** Funding acquisition (equal); project administration (equal); resources (equal); supervision (equal); writing – review and editing (equal). **Liv Beathe Gansmo:** Conceptualization (lead); data curation (equal); formal analysis (equal); methodology (equal); supervision (equal); writing – original draft (equal). **Stian Knappskog:** Conceptualization (lead); data curation (equal); formal analysis (equal); funding acquisition (lead); methodology (equal); project administration (lead); supervision (equal); writing – original draft (lead).

## FUNDING INFORMATION

This work was funded by grants from the Norwegian Cancer Society, the Norwegian Research Council, and the K.G.Jebsen Foundation.

## CONFLICT OF INTEREST

SK has received research support from Astra Zeneca, Pfizer, and Illumina, and lecture honoraria from Astra Zeneca, Pfizer, and Pierre Fabre, for all projects and activities outside of the present work. All other authors report no conflicts of interest.

## ETHICS STATEMENT

The study was approved by the Regional Committees for Ethics in Medical Research (REK Midt‐Norge and REK Vest) and analyses were performed according to the Norwegian authorities guidelines for research on human samples.

## PATIENT CONSENT STATEMENT

All samples and data were collected after obtaining written informed consent from participants.

## Supporting information


Figure S1.
Figure S2.
Figure S3.
Figure S4.
Figure S5.

Table S1.
Table S2.
Table S3.
Table S4.
Table S5.
Table S6.

## Data Availability

The data are available upon reasonable scientific request to the authors and pending project‐specific ethics approval.
