# Supplementary material for: APOBEC3A/B deletion polymorphism and endometrial cancer risk
Source: Cancer Med. 2022 Nov 16;12(6):6659–67. doi: 10.1002/cam4.5448 (PMC10067079; doi:10.1002/cam4.5448)
Supplement: Supplementary file 1 — Figure S1. Figure S2. Figure S3. Figure S4. Figure S5. [file CAM4-12-6659-s001.pptx]

## Slide 1
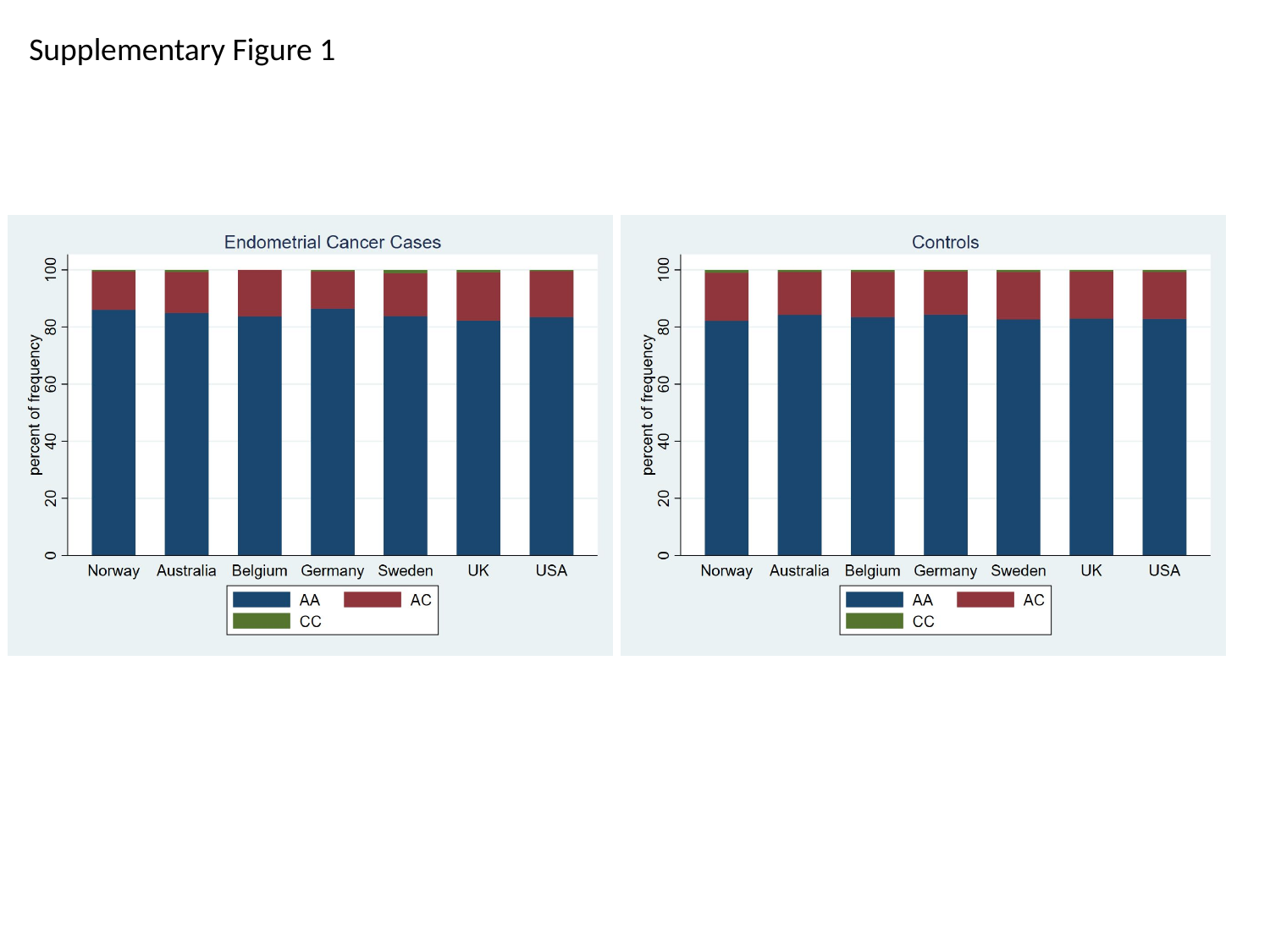

Supplementary Figure 1

## Slide 2
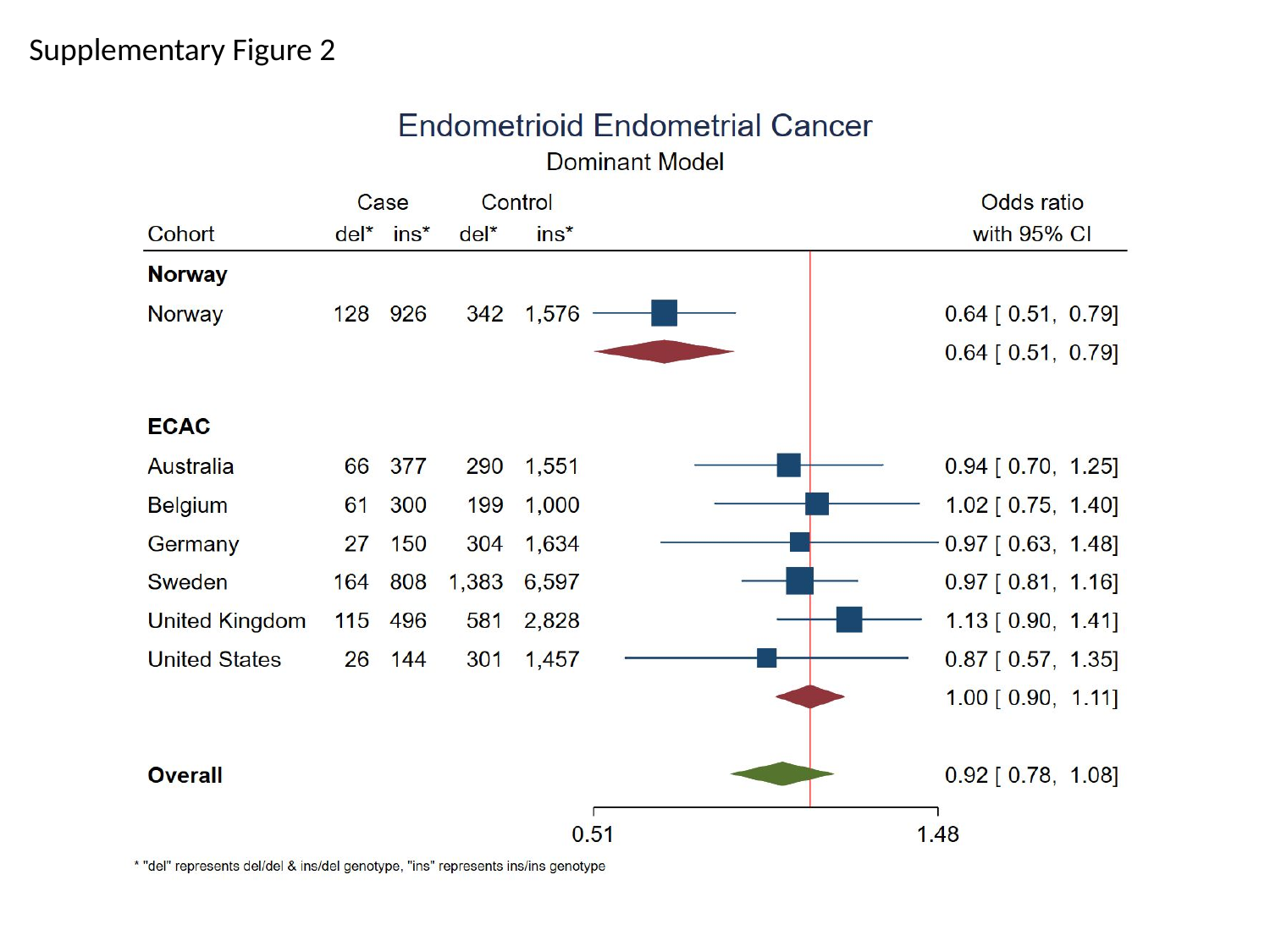

Supplementary Figure 2

## Slide 3
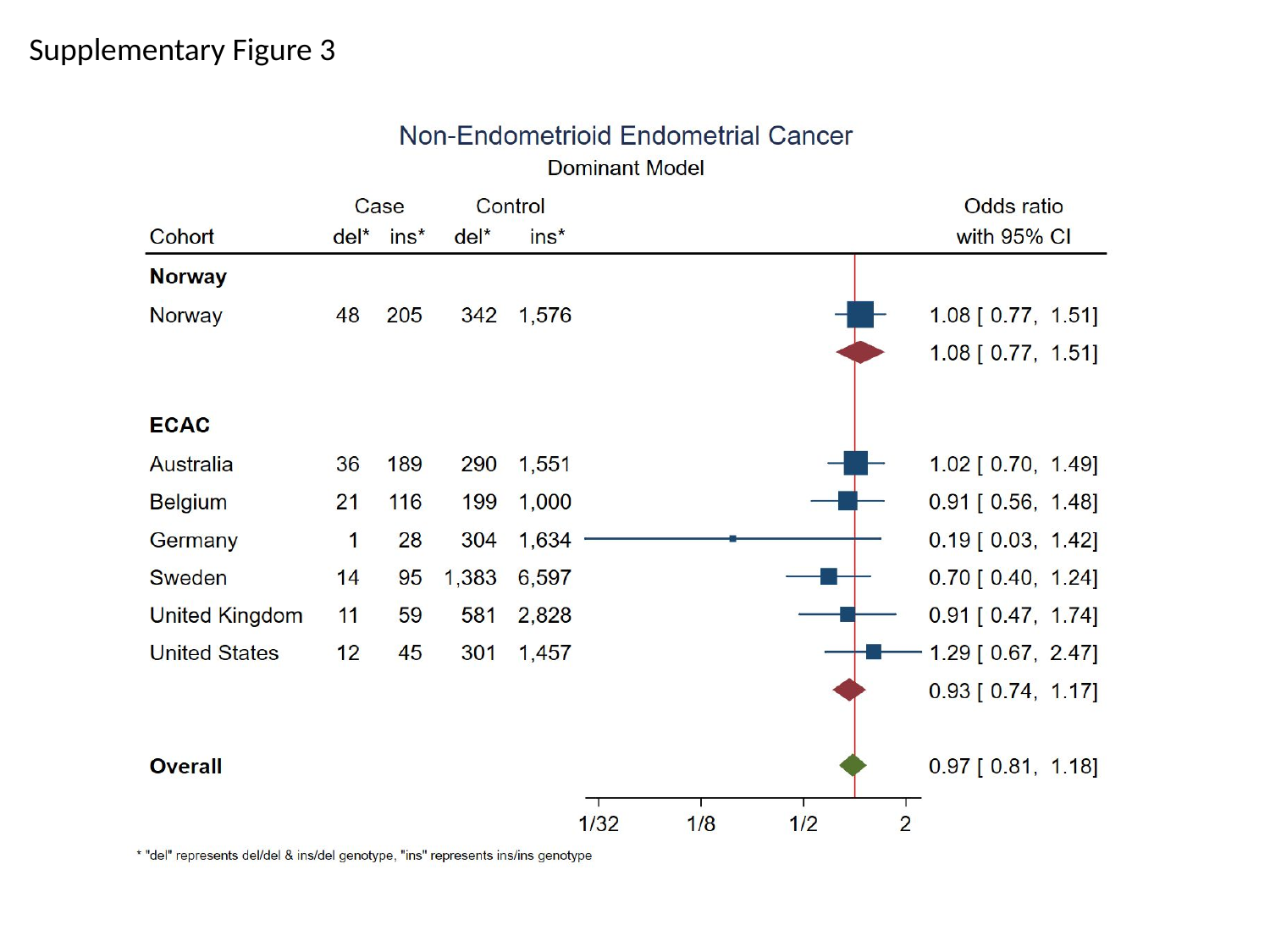

Supplementary Figure 3

## Slide 4
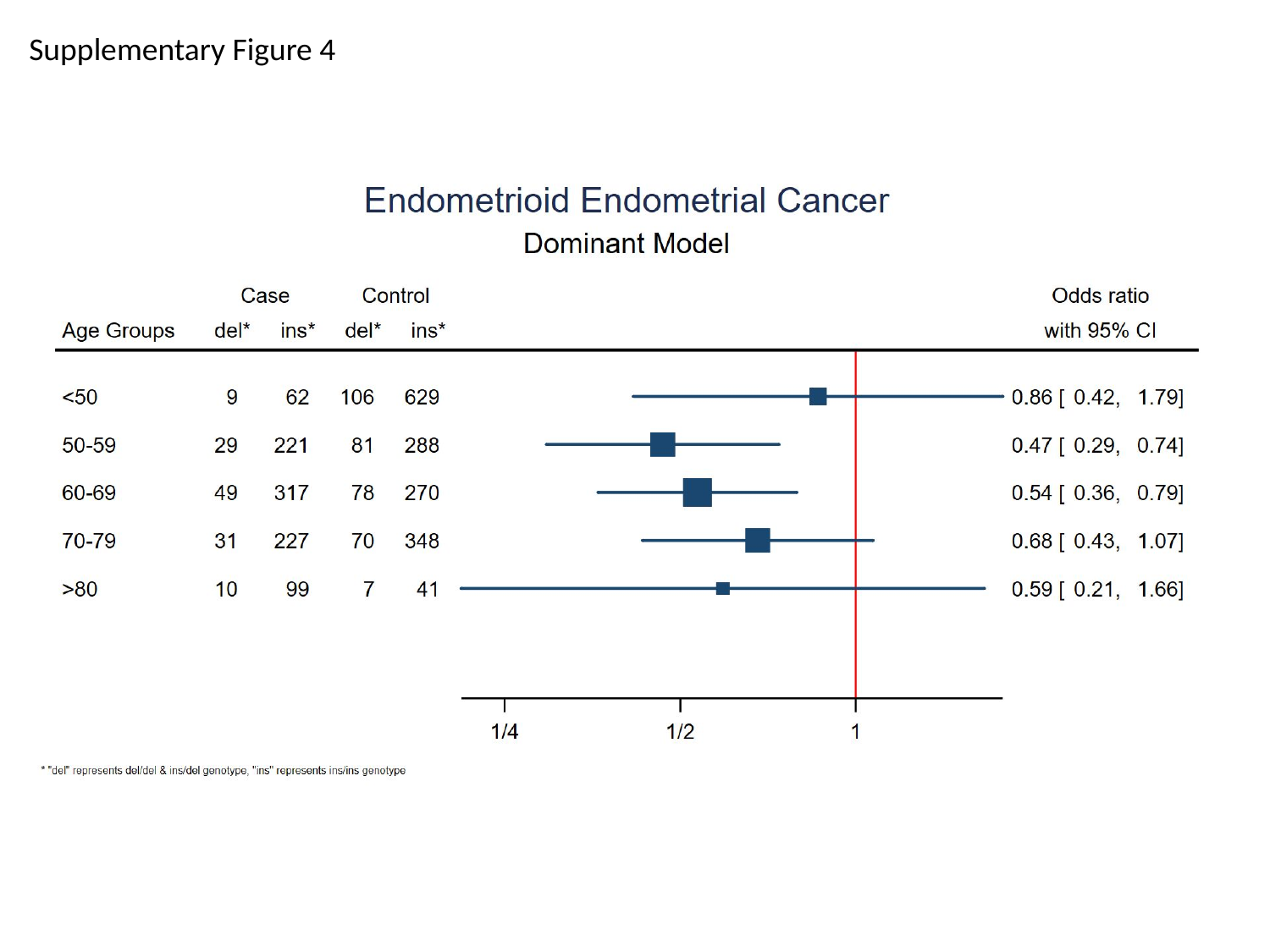

Supplementary Figure 4

## Slide 5
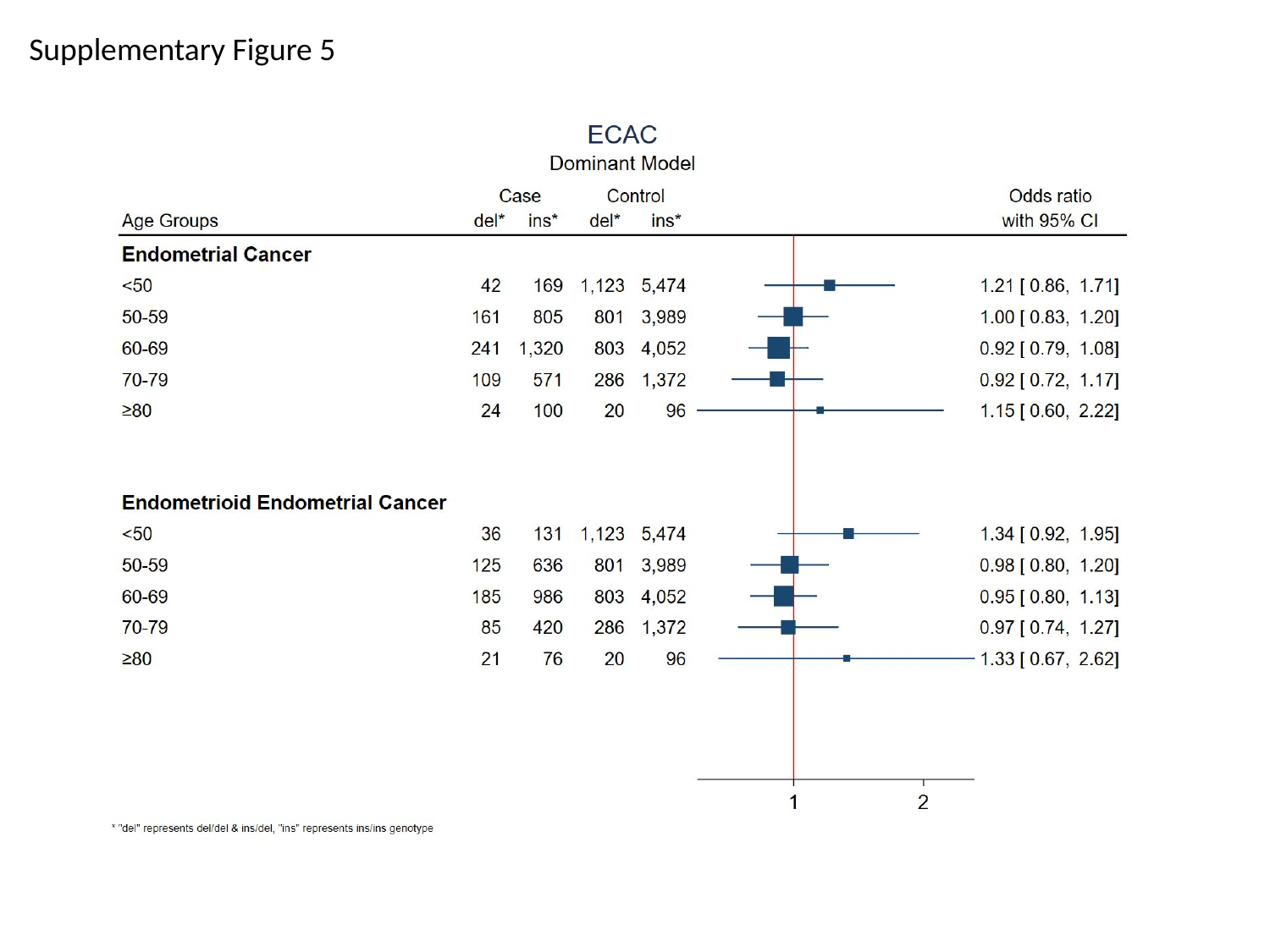

Supplementary Figure 5
