# Supplementary material for: APOBEC3A/B deletion polymorphism and endometrial cancer risk
Source: Cancer Med. 2022 Nov 16;12(6):6659–67. doi: 10.1002/cam4.5448 (PMC10067079; doi:10.1002/cam4.5448)
Supplement: Supplementary file 2 — Table S1. Table S2. Table S3. Table S4. Table S5. Table S6. [file CAM4-12-6659-s002.docx]

**Supplementary Table 1.** Summary of case-control studies assessing cancer risk associated with the *APOBEC3/B* deletion.

| **Cancer** | **Study** | **Analysis Method** | **Study Population Ethnicity** | **Sample Size (Cases/Controls)** | **Association** | **OR (CI 95%),**  **p-value** |
| --- | --- | --- | --- | --- | --- | --- |
|  |  |  |  |  |  |  |
| **Bladder** |  |  |  |  |  |  |
|  | Middlebrooks, 2016 [13] | SNP rs1014971  SNP rs1004748  SNP rs17000526 | European ancestry/ CEPH, Utah, USA | 5,832 / 10,721 | Reduced risk | 0.88 (0.84-0.93),  p=2.40E-06,  0.88 (0.84-0.93),  p=2.33E-06,  0.88 (0.84-0.93),  p=1.99E-06 |
|  | Middlebrooks, 2016 [13] | *APOBEC3A/B* deletion genotyping | European ancestry/ CEPH, Utah, USA | 1,719 / 2,566 | Reduced risk | 0,78 (0.59-0.97),  p=0.044 |
|  | Middlebrooks, 2016 [13] | *APOBEC3A/B* deletion genotyping | Asian / Japanese | 1,116 / 945 | No association | 0.87 (0.73-1.04),  p=0.128 |
|  |  |  |  |  |  |  |
|  |  |  |  |  |  |  |
| **Breast** |  |  |  |  |  |  |
|  | Komatsu, 2008 [14] | APOBEC3A/B deletion genotyping | Asian/ Japanese | 50 / 46 | No association | 3.91 (0.77-19.83), p=0.081 |
|  | Long, 2012 [15] | APOBEC3A/B deletion genotyping | Asian / Chinese | 2,623 / 1,946 | Increased risk | 1.29 (1.14-1.45), p=2.8x10^-5^ |
|  | Xuan, 2013 [16] | APOBEC3A/B deletion genotyping | European ancestry / Nashville, USA | 1,671 / 1,602 | Increased risk | 1.21 (1.02-1.43) |
|  | Rezaei, 2015 [17] | APOBEC3A/B deletion genotyping | Middle Eastern / Southeast Iran | 262/ 217 | Increased risk | 1.50 (1.03-2.19),  p=0.037 |
|  | Wen, 2016 [18] | APOBEC3B gene expression (microarray) | Asian / Chinese, Malay, Indian decent | 1,451 / 1,442 | Increased risk | 1.38 (1.10-1.74) |
|  | Revathidevi, 2016 [19] | APOBEC3A/B deletion genotyping | Asian / Indian | 224 / 478 | No association | 0.78 (0.37-1.64), p=0.847 |
|  | Marouf, 2016 [20] | APOBEC3A/B deletion genotyping | African / Moroccan | 226 / 200 | No association | 0.64 (0.34-1.21), 0.168 |
|  | Marouf, 2016 [20] | SNP rs6001376 | African / Moroccan | 226 / 200 | Increased risk | 2.15 (1.16-4.00), 0.015 |
|  | Marouf, 2016 [20] | SNP rs8142462  SNP rs28401571  SNP rs1065184 | African / Moroccan | 226 / 200 | No association | p=0.158  p=0.068  p=0.100 |
|  | Göhler, 2016 [21] | APOBEC3A/B deletion genotyping | European / Swedish | 782 / 1559 | No association | 0.70 (0.41-1.19), 0.19 |
|  | Göhler, 2016 [21] | SNP rs8142462  SNP rs2267398  SNP rs28401571  SNP rs150925968  SNP rs2142833  SNP rs6001376 | European / Swedish | 782 / 1559 | No association | 0.96 (0.53-1.72), 0.88  1.20 (0.80-1.80), 0.37  1.07 (0.70-1.64), 0.76  0.88 (0.49-1.58), 0.66  1.04 (0.67-1.63), 0.86  1.01 (0.66-1.56), 0.96 |
|  | Klonowska, 2017 [22] | APOBEC3A/B deletion genotyping | European/ Pole, Russian, Lithuanian | 2532 / 2858 | No association | 0.98 (0.83-1.15),  p=0.79 |
|  | Gansmo, 2018 [23] | APOBEC3A/B deletion genotyping | European / Norwegian | 1769 / 1918 | No association | 0.95 (0.80-1.13), 0.39 |
|  | Vitiello, 2020 [24] | APOBEC3A/B deletion genotyping | Predominantly European ancestry / Brazil | 341 / 397 | No association | 0.73 (0.50-1.06) |
|  |  |  |  |  |  |  |
| **Ovarian** |  |  |  |  |  |  |
|  |  |  |  |  |  |  |
|  | Qi, 2014 [25] | APOBEC3A/B deletion genotyping | Asian / Chinese | 1374 / 1564 | Increased risk | One-copy deletion  1.46 (1.14-1.86)  Two-copy deletion  2.53 (0.91-7.06) |
|  | Klonowska, 2017 [22] | APOBEC3A/B deletion genotyping | European/ Pole, Russian, Lithuanian | 440 / 1062 | No association | 0.75 (0.51-1.10), 0.14 |
|  | Gansmo, 2021 [26] | APOBEC3A/B deletion genotyping | European / Norwegian | 1398 / 1918 | Reduced risk | 0.75 (0.61-0.91), 0.003 |
|  |  |  |  |  |  |  |
| **Cervical** |  |  |  |  |  |  |
|  |  |  |  |  |  |  |
|  | Revathidevi, 2016 [19] | APOBEC3A/B deletion genotyping | Asian / Indian | 88 /478 | No association | 0.704 (0.235-2.113), 0.933 |
|  |  |  |  |  |  |  |
| **Oral** |  |  |  |  |  |  |
|  |  |  |  |  |  |  |
|  | Revathidevi, 2016 [19] | APOBEC3A/B deletion genotyping | Asian / Indian | 97 /478 | No association | 0.179 (0.024-1.341), 0.172 |
|  |  |  |  |  |  |  |
| **Colon** |  |  |  |  |  |  |
|  |  |  |  |  |  |  |
|  | Gansmo, 2018 [23] | APOBEC3A/B deletion genotyping | European / Norwegian | 1585 / 3827 | No association | 0.91 (0.77-1.08), 0.30 |
|  |  |  |  |  |  |  |
| **Lung** |  |  |  |  |  |  |
|  |  |  |  |  |  |  |
|  | Gansmo, 2018 [23] | APOBEC3A/B deletion genotyping | European / Norwegian | 1360 / 3827 | No association | 1.13 (0.94-1.36), 0.20 |
|  | Ben, 2021 [27] | APOBEC3A/B deletion genotyping | Asia / Chinese | 317 / 334 | Increased Risk  (two-copy deletion) | One-copy deletion  0.92 (0.65-1.30), 0.628  Two-copy deletion  2.36 (1.53-3.64), <0.001 |
|  |  |  |  |  |  |  |
| **Prostate** |  |  |  |  |  |  |
|  | Gansmo, 2018 [23] | APOBEC3A/B deletion genotyping | European / Norwegian | 2565 / 3827 | No association | 0.93 (0.78-1.10), 0.37 |
|  |  |  |  |  |  |  |

**Supplementary Table 2.** APOBEC3A/B deletion genotype as a risk predictor for endometrial cancer.

| **Cohort** | **Endometrial Cancer Risk**  **OR (95% CI) / p-value** | | | | | | |
| --- | --- | --- | --- | --- | --- | --- | --- |
|  | ***Dominant Model***  ***(ins/del + del/del vs. ins/ins)*** | ***Recessive Model***  ***(del/del vs. ins/del + ins/ins)*** | ***Co- Dominant***  ***(del/del vs. ins/ins)*** | ***Co-Dominant***  ***(ins/del vs. ins/ins)*** | | ***Additive Model***  ***(ins/ins vs. (del/del*2) + ins/del)*** | ***Allele Model***  ***(del/del*2) + ins/del) vs. (ins/del + (ins/ins*2).*** |
|  |  |  |  |  |  | |  |
| **Norway** | 0.75 (0.62-0.91)  ***p= 0.003*** | 0.48 (0.20-1.14)  *p = 0.089* | 0.46 (0.19-1.10)  *p = 0.072* | 0.77 (0.63-0.93)  ***p=0.007*** | 0.74 (0.61-0.88)  ***p=0.001*** | | 0.75 (0.63-0.90)  ***p = 0.002*** |
| **Age-adjusted** | 0.70 (0.57-0.86)  ***p= 7.16e-04*** | 0.51 (0.20-1.33) *p=0.152* | 0.28 (0.07-1.04) *p=0.052* | 0.74 (0.45-1.22) *p=0.229* | 0.64 (0.41-1.02) */ p=0.057* | | 0.65 (0.42-1.00) ***p=0.048*** |
| **Age group-adjusted** | 0.66 (0.53-0.81)  ***p=8.41e-05*** | 0.43 (0.17-1.08) *p=0.061* | 0.39 (0.15-1.00)  ***p=0.044*** | 0.67 (0.54-0.84***) p=0.0003*** | 0.64 (0.52-0.79)  ***p <0.0001*** | | 0.67 (0.55-0.81)  ***p<0.0001*** |
|  |  |  |  |  |  | |  |
| **ECAC** | 0.98 (0.89-1.07) *p=0.579* | 1.18 (0.80-1.73) *p=0.408* | 1.17 (0.79-1.72)   p= *0.424* | 0.97 (0.88-1.06) p=*0.471* | 0.98 (0.90-1.07) p=*0.690* | | 0.99 (0.91-1.08) p=0.726 |
| **Age group-adjusted** | 0.97 (0.91-1.04) *p=0.383* | 1.14 (0.82-1.59) *p=0.434* | 1.14 (0.81-1.6)  p=*0.451* | 0.96 (0.90-1.04) p=*0.303* | 0.97 (0.91-1.05) p=*0.471* | | 0.98 (0.91-1.05) p=0.508 |
| **Australia** | 0.95 (0.75-1.21) *p=0.684* | 0.95 (0.34-2.66)  *p= 0.928* | 0.95 (0.34-2.64)  p = *0.917* | 0.95 (0.74-1.22)  p= *0.692* | 0.95 (0.75-1.21) p=*0.677* | | 0.95 (0.76-1.20)  p= 0.689 |
| **Belgium^i^** | 0.98 (0.74-1.30) *p=0.907* | 0.14 (0.01-2.43)  *p= 0.067* | 0.14 (0.01-2.44)  p= *0.067* | 1.02 (0.77-1.36)  p= *0.866* | 0.95 (0.71-1.25)  p= *0.695* | | 0.94 (0.72-1.23)  p= 0.669 |
| **Germany** | 0.85 (0.56-1.28)  *p = 0.422* | 0.94 (0.12-7.38)  *p= 0.953* | 0.92 (0.12-7.21)  p*=0.935* | 0.84 (0.55-1.29)  p= *0.429* | 0.85 (0.56-1.28)  p= *0.430* | | 0.86 (0.58-1.27) p=0.449 |
| **Sweden** | 0.93 (0.78-1.10)  *p= 0.374* | 1.73 (0.94-3.18)  *p= 0.073* | 1.70 (0.93-3.12)  p= *0.084* | 0.89 (0.75-1.07)  p= *0.211* | 0.96 (0.81-1.13)  p*= 0.591* | | 0.96 (0.82-1.13)  p= 0.658 |
| **United**  **Kingdom** | 1.05 (0.90-1.23)  *p= 0.536* | 1.51 (0.74-3.06)  *p= 0.265* | 1.51 (0.75-3.08)  p*= 0.248* | 1.04 (0.88-1.22)  p*= 0.670* | 1.07 (0.91-1.24)  p=*0.420* | | 1.06 (0.92-1.23)  p= 0.420 |
| **USA** | 0.96 (0.67-1.39)  *p= 0.841* | 0.59 (0.08-4.52)  p= 0.583 | 0.59 (0.08-4.51)  p= 0.604 | 0.98 (0.67-1.42)  p= 0.916 | 0.95 (0.66-1.37)  p= 0.772 | | 0.95 (0.67-1.34)  p= 0.769 |

^i^Cohort harbors a zero-value in the *del/del* cell

Abbreviations: OR: Odds Ratio; ECAC: Endometrial Cancer Association Consortium

**Supplementary Table 3.** *APOBEC3A/B* deletion genotype as a risk factor in endometrioid endometrial cancer.

| **Cohort** | **Genotype**  **n (%)** | | | | | **Endometrioid Endometrial Cancer Risk**  **OR (95% CI) / p-value** | | | | | | | | | | | |
| --- | --- | --- | --- | --- | --- | --- | --- | --- | --- | --- | --- | --- | --- | --- | --- | --- | --- |
|  | ***ins/ins*** | ***ins/del*** | | | ***del/del*** | ***Dominant Model***  ***(ins/del + del/del vs. ins/ins)*** | ***Recessive Model***  ***(del/del vs. ins/del + ins/ins)*** | | ***Co- Dominant***  ***(del/del vs. ins/ins)*** | | ***Co-Dominant***  ***(ins/del vs. ins/ins)*** | | ***Additive Model***  ***(ins/ins vs. (del/del*2) + ins/del)*** | | ***Allele Model***  ***(del/del*2) + ins/del) vs. (ins/del + (ins/ins*2).*** | | |
|  |  | | | | |  |  | |  | |  | |  | |  | | |
| **Norway** | 926 (87.9) | 124 (11.8) | | | 4 (0.38) | 0.64 (0.51-0.79)  ***p = 3.55e-05*** | 0.38 (0.13-1.12)  *p = 0.054* | | 0.36 (0.12-1.06)  p = 0.063 | | 0.65 (0.52-0.82)  **p <0.0001** | | 0.62 (0.50-0.77) **p <0.0001** | | 0.64 (0.52-0.79)  p*=* ***3.152e-05*** | | |
| **Age-adjusted** |  |  | | |  | 0.63 (0.50-0.79***)***  ***p=4.10e-05*** | 0.41 (0.13-1.23) *p=0.084* | | 0.18 (0.04-0.86) ***p=0.015*** | | 0.71 (0.42-1.18) *p=0.181* | | 0.60 (0.37-0.96) ***p=0.032*** | | 0.61 (0.39-0.95***) p=0.026*** | | |
| **Age group-adjusted** |  |  | | |  | 0.57 (0.46-0.72)  ***p=1.72e-06*** | 0.33 (0.11-0.99*)* ***p=0.029*** | | 0.30 (0.10-0.91)  ***p= 0.024*** | | 0.60 (0.47-0.75) ***p<0.0001*** | | 0.56 (0.49-0.71)  ***p <0.0001*** | | 0.59 (0.47-0.73) ***p<0.0001*** | | |
|  |  | | | | |  |  | |  | |  | |  | |  | | |
| **ECAC** | 2275 (83.2) | | 439 (16.1) | 20 (0.73) | | 1.00 (0.90 -1.11)  *p=0.726* | | 1.12 (0.69-1.79)  *p=0.653* | | 1.11 (0.69-1.79) *p=0.659* | | 0.99 (0.89-1.10) *p=0.847* | | 1.00 (0.90-1.11)  *p= 0.978* | | 1.00 (0.90-1.11) *p= 0.992* |  |
| **Age group-adjused** |  | |  |  | | 0.99 (0.90-1.11)  *p=0.949* | | 1.23 (0.77-1.98)  *P=0.386* | | 1.23 (0.77-1.98)  *p=0.386* | | 0.99 (0.89-1.10)  *p=0.825* | | 1.00 (0.90-1.12)  *p=0.931* | | 1.01 (0.91-1.11)  *p=0.912* |  |
| **Australia** | 377 (85.1) | | 64 (14.4) | 2 (0.45) | | 0.94 (0.70-1.25)  *p= 0.656* | | 0.59 (0.13-2.61)  *p= 0.484* | | 0.59 (0.13-2.60) *p=0.478* | | 0.95 (0.71-1.28)  *p= 0.754* | | 0.92 (0.69 -1.22) *p= 0.569* | | 0.92 (0.70-1.21) *p= 0.570* |  |
| **Belgium^i^** | 300 (83.1) | | 61 (16.9) | 0 (0) | | 1.02 (0.75-1.40)  *p=0.893* | | 0.19 (0.01-3.37) *p=0.120* | | 0.20 (0.01-3.40) *p=0.122* | | 1.06 (0.78-1.46)  *p=0.698* | | 0.98 (0.72-1.34) *p=0.911* | | 0.98 (0.72-1.32) *p=0.877* |  |
| **Germany** | 150 (84.7) | | 26 (14.7) | 1 (0.56) | | 0.97 (0.63-1.48)  *p=0.880* | | 1.10 (0.14-8.61) *p=0.931* | | 1.09 (0.14-8.57) *p= 0.935* | | 0.96 (0.62-1.49)  *p= 0 .866* | | 0.97 (0.64-1.48) *p= 0.893* | | 0.97 (0.65-1.46) *p= 0.899* |  |
| **Sweden** | 808 (83.1­) | | 151 (15.5) | 13 (1.34) | | 0.97 (0.81-1.16)  *p= 0.721* | | 1.95 (1.06-3.59) ***p=0.028*** | | 1.93 (1.05-3.55) ***p=0.031*** | | 0.93 (0.77-1.12)  *p= 0.427* | | 1.00 (0.85-1.19) *p=0.955* | | 1.01 (0.86-1.19) *p= 0.890* |  |
| **United**  **Kingdom** | 496 (81.2) | | 111 (18.2) | 4 (0.65) | | 1.13 (0.90-1.41)  *p= 0.285* | | 1.18 (0.40-3.47) *p=0.769* | | 1.20 (0.41-3.54) *p=0.741* | | 1.13 (0.90-1.41)  *p=0.300* | | 1.13 (0.91-1.41) *p= 0.270* | | 1.12 (0.91-1.37) *p= 0.290* |  |
| **USA^i^** | 144 (84.7) | | 26 (15.3) | 0 (0) | | 0.87 (0.57-1.35)  *p=0.544* | | 0.38 (0.02-6.41)  *p= 0.261* | | 0.37 (0.02-6.32) *p=0.257* | | 0.91 (0.59-1.41) *p=0.684* | | 0.84 (0.54-1.29) *p=0.425* | | 0.84 (0.56-1.28) *p=0.425* |  |

^i^Cohort harbors a zero-value in the *del/del* cell

Abbreviations: OR: Odds Ratio; ECAC: Endometrial Cancer Association Consortium.

**Supplementary Table 4.** *APOBEC3A/B* deletion genotype as a risk factor in non-endometrioid endometrial cancer.

| **Cohort** | **Genotype**  **n (%)** | | | | | **Non-Endometrioid Endometrial Cancer Risk**  **OR (95% CI) / p-value** | | | | | |
| --- | --- | --- | --- | --- | --- | --- | --- | --- | --- | --- | --- |
|  | ***ins/ins*** | | ***ins/del*** | | ***del/del*** | ***Dominant Model***  ***(ins/del + del/del vs. ins/ins)*** | ***Recessive Model***  ***(del/del vs. ins/del + ins/ins)*** | ***Co- Dominant***  ***(del/del vs. ins/ins)*** | ***Co-Dominant***  ***(ins/del vs. ins/ins)*** | ***Additive Model***  ***(ins/ins vs. (del/del*2) + ins/del)*** | ***Allele Model***  ***(del/del*2) + ins/del) vs. (ins/del + (ins/ins*2).*** |
|  |  | | | | |  |  |  |  |  |  |
| **Norway** | 205 | | 46 | | 2 | 1.08 (0.77-1.51)  *p = 0.657* | 0.80 (0.18-3.44)  *p = 0.760* | 0.81 (0.19-3.50)  *p = 0.777* | 1.09 (0.78-1.54)  *p = 0.603* | 1.06 (0.77-1.48) /  *p = 0.709* | 1.06 (0.77-1.44) /  *p = 0.734* |
| **Age-adjusted** |  | |  | |  | 1.08 (0.76-1.53)  *p=0.679* | 0.98 (0.22-4.41) *p=0.975* | 0.57 (0.14-2.29) *p=0.425* | 1.11 (0.55-2.27) *p=0.769* | 0.97 (0.54-1.79)/ *p=0.945* | 0.95 (0.54-1.66)/ *p=0.852* |
| **Age group-adjusted** |  | |  | |  | 0.98 (0.69-1.40)  *p=0.912* | 0.82 (0.18-3.68) *p=0.789* | 0.81 (0.19-3.56) *p=0.782* | 0.99 (0.69-1.43) *p=0.956* | 0.97 (0.68-1.39)/ *p=0.869* | 0.97 (0.70-1.35)/ *p=0.871* |
|  |  | | | | |  |  |  |  |  |  |
| **ECAC** | 532 | 91 | | 4 | | 0.93 (0.74-1.17)  *p=0.258* | 0.97 (0.36-2.64) *p=0.955* | 0.95 (0.35-2.59) *p=0.923* | 0.88 (0.70-1.10)  *p= 0.254* | 0.88 (0.71-1.10)  *p= 0.261* | 0.89 (0.72-1.10)  *p= 0.284*` |
| **Age group-adjusted** |  |  | |  | | 0.88 (0.71-1.10)  *p=0.271* | 1.06 (0.38-2.94)  *p=0.907* | 1.03 (0.37-2.87)  *p=0.947* | 0.88 (0.70-1.10)  *p=0.255* | 0.89 (0.71-1.11)  *p=0.287* | 0.90 (0.73-1.11)  *p=0.313* |
| **Australia** | 189 | 33 | | 3 | | 1.02 (0.70-1.49) /  p = 0.923 | 1.76 (0.50-6.18) / p= 0.369 | 1.76 (0.50-6.18) / p= 0.372 | 0.98 (0.66-1.45) / p= 0.924 | 1.05 (0.73-1.52) /  p= 0.783 | 1.05 (0.74-1.49) / p=0.766 |
| **Belgium^i^** | 116 | 21 | | 0 | | 0.91 (0.56-1.48) /  p=0.705 | 0.51 (0.03-8.88) /  p=0.338 | 0.51 (0.03-8.81) /  p=0.336 | 0.95 (0.58-1.55)/ p=0.830 | 0.87 (0.54-1.43)/  p=0.590 | 0.88 (0.55-1.40)/  p=0.587 |
| **Germany^i^** | 28 | 1 | | 0 | | 0.19 (0.03-1.42) /  p=0.071 | 3.11 (0.18-54.37)/  p= 0.698 | 2.73 (0.16-47.74)/ p=0.679 | 0.20 (0.03-1.46)/ p=0.078 | 0.19 (0.03-1.37)/  p=0.064 | 0.20 (0.03-1.44)/  p=0.076 |
| **Sweden^i^** | 95 | 14 | | 0 | | 0.70 (0.40-1.24) /  p=0.218 | 0.65 (0.04-10.62) / p=0.385 | 0.62 (0.04-10.15) / p=0.374 | 0.73 (0.42-1.29)/  p=0.277 | 0.68 (0.38-1.19) / p=0.171 | 0.69 (0.40-1.19) / p=0.184 |
| **United**  **Kingdom^i^** | 59 | 11 | | 0 | | 0.91 (0.47-1.74) /  p=0.770 | 1.23 (0.07-20.63) /  p=0.531 | 1.22 (0.07-20.43) /  p= 0.529 | 0.94 (0.49-1.80) / p=0.847 | 0.88 (0.46-1.68)/  p=0.696 | 0.88 (0.47-1.64)/  p=0.696 |
| **USA** | 45 | 11 | | 1 | | 1.29 (0.67-2.47) /  p= 0.439 | 2.40 (0.31-18.64) / p= 0.389 | 2.49 (0.32-19.45) / p= 0.368 | 1.24 (0.63-2.42) / p= 0.534 | 1.34 (0.71-2.51)/  p= 0.359 | 1.31 (0.73-2.37) /  p=0.364 |

^i^Cohort harbors a zero-value in the *del/del* cell

Abbreviations: OR: Odds Ratio; ECAC: Endometrial Cancer Association Consortium.

**Supplementary Table 5.** Distribution of *APOBEC3A/B* deletion genotype in subtypes (endometrioid and non-endometrioid) of endometrial cancer in the Norwegian cohort.

| **Histology** | **Genotype n (%)** | | |
| --- | --- | --- | --- |
|  | ***ins/ins*** | ***ins/del*** | ***del/del*** |
|  |  |  |  |
| **Endometrioid** | 926 (87.9) | 124 (11.8) | 4 (0.38) |
| **Serous** | 95 (79.8) | 23 (19.3) | 1 (0.84) |
| **Clear Cell** | 36 (80) | 8 (17.8) | 1 (2.22) |
| **Adenosquamous** | 7 (63.6) | 4 (36.4) | 0 (0) |
| **Carcinosarcoma** | 45 (88.2) | 6 (11.8) | 0 (0) |
| **Undifferentiated/Other** | 22 (81.5) | 5 (18.5) | 0 (0) |

**Supplementary Table 6.** Age at diagnosis and risk of endometrial cancer in patients with *APOBEC3A/B* deletion genotype.

| **Cohort** | **Genotype**  **n (%)** | | | **Cancer Risk**  **OR (95% CI) / p-value** | | | | | |
| --- | --- | --- | --- | --- | --- | --- | --- | --- | --- |
|  | ***ins/ins*** | ***ins/del*** | ***del/del*** | ***Dominant Model***  ***(ins/del + del/del vs. ins/ins)*** | ***Recessive Model***  ***(del/del vs. ins/del + ins/ins)*** | ***Co- Dominant***  ***(del/del vs. ins/ins)*** | ***Co-Dominant***  ***(ins/del vs. ins/ins)*** | ***Additive Model***  ***(ins/ins vs. (del/del*2) + ins/del)*** | ***Allele Model***  ***(del/del*2) + ins/del) vs. (ins/del + (ins/ins*2).*** |
| **Endometrial Cancer** |  |  |  |  |  |  |  |  |  |
| **<50^i^** | 66 (86.8) | 10 (13.2) | 0 (0) | 0.90 (0.45-1.80)  *p = 0.762* | 1.06 (0.06-19.92) *p = 0.519* | 1.05 (0.06-19.75)  *p = 0.517* | 0.93 (0.47-1.88) *p= 0.849* | 0.87 (0.43-1.74) *p = 0.686* | 0.87 (0.45-1.70) *p = 0.685* |
| **50-59** | 245 (87.5) | 32 (11.4) | 3 (1.07) | 0.51 (0.33-0.78) ***p = 0.002*** | 0.56 (0.14-2.19) *p= 0.398* | 0.50 (0.13-1.97) *p = 0.315* | 0.51 (0.32-0.80) ***p = 0.003*** | 0.51 (0.33-0.77) ***p = 0.001*** | 0.54 (0.36-0.80) ***p = 0.002*** |
| **60-69** | 388 (84.9) | 67 (14.7) | 2 (0.44) | 0.62 (0.43-0.88) ***p = 0.008*** | 0.30 (0.06-1.56) *p = 0.130* | 0.28 (0.05-1.45) *p = 0.104* | 0.64 (0.44-0.92) ***p = 0.016*** | 0.60 (0.42-0.85)  ***p = 0.004*** | 0.62 (0.45-0.87) ***p = 0.005*** |
| **70-79** | 296 (87.1) | 43 (12.6) | 1 (0.29) | 0.74 (0.49-1.11) *p = 0.145* | 0.41 (0.04-3.94) *p = 0.423* | 0.39 (0.04-3.79) *p = 0.402* | 0.75 (0.50-1.14) *p = 0.181* | 0.72 (0.48-1.08) *p = 0.116* | 0.74 (0.50-1.09) *p = 0.126* |
| **≥80^i^** | 138 (88.5) | 18 (11.5) | 0 (0) | 0.76 (0.30-1.96) *p = 0.574* | NA* | NA* | 0.76 (0.30-1.96) *p = 0.574* | 0.76 (0.30-1.96) *p = 0.574* | 0.78 (0.32-1.92) *p = 0.586* |
|  |  |  |  |  |  |  |  |  |  |
| **Endometrioid Endometrial Cancer** |  |  |  |  |  |  |  |  |  |
| **<50^i^** | 62 (87.3) | 9 (12.7) | 0 (0) | 0.86 (0.42-1.79) *p=0.684* | 1.14 (0.06-21.33) *p=0.533* | 1.12 (0.06-21.03) *p=0.530* | 0.90 (0.43-1.86) *p=0.766* | 0.83 (0.40-1.72) *p=0.616* | 0.84 (0.41-1.69) *p=0.618* |
| **50-59** | 221 (88.4) | 27 (10.8) | 2 (0.80) | 0.47 (0.29-0.74) ***p=0.0007*** | 0.42 (0.09-2.02) *p=0.263* | 0.37 (0.08-1.81) *p=0.203* | 0.48 (0.30-0.76) ***p=0.0018*** | 0.46 (0.29-0.72) ***p=0.0005*** | 0.49 (0.32-0.75) ***p=0.0008*** |
| **60-69** | 317 (86.6) | 48 (13.1) | 1 (0.27) | 0.54 (0.36-0.79) ***p=0.0016*** | 0.19 (0.02-1.62) *p=0.089* | 0.17 (0.02-1.47) *p=0.068* | 0.56 (0.38-0.83) ***p=0.004*** | 0.51 (0.35-0.76) ***p=0.0006*** | 0.54 (0.37-0.78) ***p= 0.0009*** |
| **70-79** | 227 (88.0) | 30 (11.6) | 1 (0.39) | 0.68 (0.43-1.07) *p=0.094* | 0.54 (0.06-5.20) *p=0.587* | 0.51 (0.05-4.94) *p=0.555* | 0.69 (0.43-1.09) *p=0.109* | 0.67 (0.43-1.05) *p=0.081* | 0.69 (0.45-1.06) *p=0.091* |
| **≥80^i^** | 99 (90.8) | 10 (9.17) | 0 (0) | 0.59 (0.21-1.66) *p=0.315* | NA* | NA* | 0.59 (0.21-1.66) *p=0.315* | 0.61 (0.23-1.66) *p=0.315* | 0.61 (0.23-1.66) *p=0.329* |

^i^Cohort harbors a zero-value in the *del/del* cell

* Calculation is not applicable due to the two zero-counts in del/del genotype in cases and controls.

Abbreviations: OR: Odds Ratio.
